# Changes in Support for Advance Provision and Over-the-Counter Access to Medication Abortion

**DOI:** 10.1001/jamanetworkopen.2024.54767

**Published:** 2025-01-16

**Authors:** M. Antonia Biggs, Rosalyn Schroeder, Shelly Kaller, Daniel Grossman, Karen A. Scott, Lauren J. Ralph

**Affiliations:** 1 Advancing New Standards in Reproductive Health, University of California, San Francisco, Oakland; 2 Birthing Cultural Rigor, LLC, Nashville, Tennessee

## Abstract

**Importance:**

Since *Dobbs v Jackson Women’s Health Organization* (*Dobbs*) removed federal abortion protections, people’s views about alternative models of abortion care may have been impacted, yet research on this topic is limited.

**Objective:**

To examine changes in national support for and personal interest in advance provision (AP) and over-the-counter (OTC) access to medication abortion.

**Design, Setting, and Participants:**

Two nationally representative cross-sectional online surveys were administered to a market research firm’s panel members who were assigned female at birth (AFAB) and aged 15 to 49 years from December 2021 to January 2022 (before *Dobbs*) and June to July 2023 (after *Dobbs*). Data were analyzed from February 2023 to June 2024.

**Exposure:**

Completion of survey before and after *Dobbs*.

**Main Outcomes and Measures:**

Changes from before to after *Dobbs* in 4 primary outcome measures were examined: support for and personal interest in AP and OTC access to medication abortion.

**Results:**

A total of 6982 AFAB people before *Dobbs* and 3561 after *Dobbs* completed at least 1 of 4 primary outcome measures. From before to after *Dobbs*, 2666 (weighted 31.3%) and 1258 (weighted 30.1%) were aged 30 to 39 years, 1395 (21.4%) and 708 (21.5%) reported their race and ethnicity as Hispanic/Latinx, 594 (13.7%) and 304 (13.6%) as Black non-Hispanic/Latinx, and 4504 (54.6%) and 2270 (54.2%) as White non-Hispanic/Latinx. There was a significant increase from before to after *Dobbs* in national support for AP (48.9% before; 95% CI, 47.1% to 50.6%; 55.1% after; 95% CI, 52.8% to 57.3%) and OTC access (49.4% before; 95% CI, 47.6% to 51.1%; 55.2% after; 95% CI, 52.9% to 57.5%) and an increase in personal interest in AP (23.6% before; 95% CI, 22.2% to 25.1%; 26.4% after; 95% CI, 24.3% to 28.4%) and OTC access (36.0% before; 95% CI, 34.3% to 37.6%; 42.5% after; 95% CI, 40.2% to 44.7%). Among people living in states with abortion bans, larger increases in personal interest in AP (5.3 percentage points [pp]; 95% CI, 0.5 to 10.3 pp) and OTC access (9.4 pp; 95% CI, 3.9 to 14.9 pp) were observed than among people in states without bans (1.4 pp; 95% CI, −1.7 to 4.6 pp and 5.4 pp; 95% CI, 2.0 to 8.9 pp, respectively).

**Conclusions and Relevance:**

In this serial cross-sectional analysis of people aged 15 to 49 years before *Dobbs *and 1 year after *Dobbs*, findings suggested that national support for expanded access to medication abortion has grown. Alternative models of care, such as AP and OTC, have the potential to offer a promising approach to abortion care, particularly for people living in abortion-restricted states.

## Key Points

**Question:**

Has national support for and personal interest in advance provision (AP) and over-the-counter (OTC) access to medication abortion changed since the removal of federal abortion protections due to the *Dobbs v Jackson Women’s Health *(*Dobbs*) decision?

**Findings:**

In this cross-sectional study among individuals assigned female at birth surveyed before *Dobbs* (6982 participants) and after *Dobbs* (3562 participants), national support for AP and OTC access and personal interest in OTC access to medication abortion increased significantly from before to after Dobbs, although personal interest in AP access did not change significantly. Respondents in states with greater abortion restrictions were significantly more likely to report increases in support for and interest in both AP and OTC access from before to after *Dobbs* compared with those in less restrictive states.

**Meaning:**

These findings suggest growing national support for expanded access to medication abortion, particularly among people living in abortion-restricted states.

## Teaser

This cross-sectional study evaluates changes in national support for and personal interest in advance provision and over-the-counter access to medication abortion in the US since the *Dobbs v Jackson Women’s Health* Supreme Court decision.

## Introduction

Since the US Supreme Court eliminated the federal right to abortion in *Dobbs v Jackson Women’s Health Organization* (*Dobbs*) in June 2022, many states implemented total or near total bans on abortion, dramatically shifting access to abortion in the US.^1,2^ Furthermore, since the Food and Drug Administration (FDA) lifted the in-person dispensing requirement for mifepristone in April 2021 due to the COVID-19 pandemic, people have been increasingly accessing medication abortion through telehealth and online models of care, where patients report their medical history to determine their eligibility for medication abortion (ie, pregnancy duration or contraindications) and receive the medications in the mail.^3,4^ As of March 2024, 20% of all abortions within the formal health care sector in the US were through telehealth.^2^ Medication abortion is very safe and effective,^5,6^ including when accessed without an in-person visit, and has the potential to further fill in gaps in care.^7,8,9,10,11,12,13,14^

Advance provision (AP) and over-the-counter (OTC) access to medication abortion are 2 additional models of care that have the potential to further increase access to abortion. In the AP model, people receive mifepristone and misoprostol medications in advance of a pregnancy from a clinician for later use. Although not widely available, the AP model can be accessed from some telehealth practitioners.^15^ Emerging research indicates that the AP model is feasible, including for young people, and that people mostly chose it due to personal choice, legal restrictions, and privacy, although requestors tend to be overrepresented by people living in urban and higher income communities.^15,16,17,18,19,20^ The OTC model represents another promising yet currently unavailable approach. With OTC access, people could obtain the medications at a local pharmacy or other retailer without a prescription. Evidence suggests that medication abortion meets many of the FDA criteria for an OTC switch.^21^ Although unlikely to be sold OTC in retail stores in states with abortion bans, both AP and OTC models have the potential to reduce travel and ensure earlier access to abortion care for people in states with and without bans, and unlike the telehealth provision, these 2 models do not require internet access or a mailing address, which may pose a barrier for some people.

In our earlier work surveying a national US sample of self-identified adult women in 2017, 44% supported and 32% were personally interested in AP, and 37% supported and 23% were personally interested in OTC access.^22^ Personal interest in these 2 models of care was even higher among people experiencing obstacles to reproductive health care services and among abortion patients.^18,19,20,22,23,24^ The current study sought to assess national changes in people’s interest and support for AP and OTC access to medication abortion between the periods shortly before and after the *Dobbs* decision among a national sample of people who self-reported being assigned female at birth (AFAB), including young people.

## Methods

### Study Design

We fielded 2 serial, cross-sectional, nationally representative online surveys before *Dobbs* (December 2021 to January 2022) and after *Dobbs* (June to July 2023), to people aged 15 to 49 years. A market research firm administered the survey to their panel members using probability-based sampling techniques so that when applying survey weights, the sample would be representative of the noninstitutionalized, English- and Spanish-speaking population living in the US according to US census data. For this study, panel members aged 15 to 49 years who spoke English or Spanish were eligible. Reminders to nonresponders were sent 3 and 8 days following the initial survey invitation. This study followed the Strengthening the Reporting of Observational Studies in Epidemiology (STROBE) reporting guideline for cross-sectional studies.^25^ Participants and the parents of minors (aged 15-17 years) provided electronic informed consent. Participants were reimbursed through the market research firm’s points program in cash-equivalent checks in amounts reflecting their level of panel participation (approximately $4 to $6 per month). The University of California, San Francisco institutional review board approved this study. Study details have been published elsewhere.^26^

Our sampling strategy differed slightly by survey wave. Both surveys included over 7000 AFAB participants. We powered the study to estimate the prevalence of self-managed abortion, a fairly rare outcome.^27^ Questions related to interest in and support for alternative models of medication abortion provision were administered to the entire pre-*Dobbs* AFAB sample (7016 participants) and a random sample of half the post-*Dobbs* sample (3576 participants), which provided sufficient power to test differences over time in these outcomes.

To develop the pre-*Dobbs* survey, we first cognitively tested previously published items^22^ related to interest and support for AP and OTC with 9 panel members aged 25 to 45 years. During interviews we assessed whether items captured their intended meaning and revised items accordingly. Also, throughout the course of the study, we engaged an advisory board of reproductive health and justice leaders and clinicians who, in their professional roles, represent the lived experiences of people who may lack access to abortion. The advisory board provided input on the study design and dissemination activities, including insight into the reception of our research findings among the communities they serve.

### Outcome Variables

We examined responses to 4 questions regarding people’s personal interest in and support for AP and OTC access. To assess personal interest in AP and OTC, after describing each model (see eFigure in Supplement 1), we asked “Would you be interested in getting abortion pills [ahead of time from a doctor just in case?] and [over the counter from a pharmacy without a prescription if you were pregnant and didn’t want to be?]”, with 5-point Likert scale options. We coded “definitely yes” and “probably yes” as personally interested in AP or OTC and “definitely no,” “probably no,” and “don’t know” as not interested.

To assess support for AP and OTC, we then asked “Even if you are not interested in this option for yourself, would you be in favor of or opposed to people being able to get abortion pills [ahead of time from a doctor just in case?] and [over the counter from a pharmacy without a prescription?]” and offered participants 5-point Likert scale options. We coded “I am in favor” and “I am somewhat in favor” as supportive and “I am opposed,” “I am somewhat opposed,” and “Not sure” as not supportive. We also asked people to select advantages and disadvantages of the AP and OTC models from a predefined list of options reported in the prior literature,^22^ including an open-ended other category. Open-text responses were recoded into the existing close-ended responses.

### Participant Demographic Characteristics, Health History, and Experiences

We examined differences in changes in interest in AP and OTC by participant sociodemographic characteristics using variables provided by the market research firm, including self-reported age; self-reported race and ethnicity (Asian or Pacific Islander non-Hispanic/Latinx, Black non-Hispanic/Latinx, Hispanic/Latinx, White non-Hispanic/Latinx, more than 1 race or other race [included American Indian or Alaska Native, people who selected more than 1 race, and people who selected other]); language that the participant used to completed the survey; identifying as lesbian, gay, bisexual, queer, transgender, nonbinary, gender nonconforming, intersex, or other; political party; religion; living below the federal poverty level (FPL); living in a metropolitan statistical area (MSA); and geographic region. We included race, ethnicity, and language as a proxy for experiences of racism, which may influence people's views about how to access abortion care. For minors, these variables relied on parents’ reports of income and state.^28^ We created a categorical variable that combined self-reported race and Hispanic ethnicity and survey language, which included all race and Hispanic ethnicity survey options exactly as they appeared in the survey, with the exception of Native Hawaiian/Pacific Islander (7 participants) and Asian (372 participants), which were grouped into an Asian or Pacific Islander category and American Indian or Alaska Native (36 participants), and people who selected more than 1 race (352 participants) were included in a more than 1 race or other category. Anyone who self-reported Hispanic ethnicity was coded in the Hispanic ethnicity category, which we divided by whether they completed the survey in English or Spanish, as a proxy for acculturation. We also examined whether people lived in a state with a total or near ban on abortion (6- to 18-week ban) as of July 2023, according to their state of residence, and their health history and health care experiences, including history of pregnancy and abortion and experiences of medical mistreatment (participant indicated that when seeking health care, doctors, nurses, or other providers ever made them feel their symptoms were not real, not severe, or not important, or they felt ridiculed or humiliated by doctors, nurses, or other providers). Lastly, we assessed the number of barriers experienced trying to access reproductive health care (RH) services in the past 3 years, where RH services were defined as a Pap smear, which is a test to check for cervical cancer, or family planning, like birth control methods. Those who had accessed RH services selected any from a list of 10 potential barriers, which included whether they experienced difficulty finding a clinic offering RH services, finding a clinic that accepted their insurance, finding a clinic where they felt comfortable, finding services where people spoke their language, finding transportation, finding childcare, getting time off work, paying for services, getting to a clinic without telling people they did not want to tell, and getting to a clinic when a partner or family member did not want them to go.

### Statistical Analysis

For all analyses, we used survey weights designed to produce estimates representative of the noninstitutionalized US population, based on US Census data and any differential nonresponse. We conducted descriptive analyses of people’s interest in and support for AP and OTC models before (2021-2022) and after (2023) *Dobbs*. We conducted unadjusted and adjusted logistic regression analyses using group by time interactions and marginal contrasts to assess changes in interest in AP and OTC access over time by participant characteristics. Adjusted analyses controlled for the participant sociodemographic characteristics, health history, and health care experiences described previously. We accounted for clustering for the 1709 participants who completed the survey in both survey years using cluster-robust standard errors. All analyses were conducted in Stata version 18 (StataCorp). Significance was reported at a 2-sided *P* ≤ .05. Due to missing data on all 4 outcomes, we excluded 35 out of 7016 participants in 2021 to 2022 and 15 out of 3576 who were administered outcome questions in 2023 from all analyses. To handle missing covariate data, which was very low (0%-.6%), we employed casewise deletion methods. Data were analyzed from February 2023 to June 2024.

## Results

A total of 28 417 (15 345 in 2021-2022 and 13 072 in 2023) adult AFAB panel members were invited to participate, of which 14 674 (7388 in 2021-2022 and 7286 in 2023) completed the eligibility screener. Of the 14 454 eligible, 13 626 completed the survey (6841 in 2021-2022 and 6785 in 2023). Among the 764 people ages 17 years and under eligible, 538 completed the survey (175 in 2021-2022 and 363 in 2023). Our final analytic sample included the 10 543 (6982 in 2021-2022 and 3561 in 2023) observations which contained primary outcome data.

Participant demographic and health characteristics are presented in Table 1. From before to after *Dobbs*, 2666 (weighted 31.3%) and 1258 (30.1%) were aged 30-39 years, 1395 (21.3%) and 708 (21.4%) reported their race and ethnicity as Hispanic/Latinx, 594 (13.7%) and 304 (13.6%) as Black non-Hispanic/Latinx, and 4504 (54.6%) and 2270 (54.2%) as White non-Hispanic/Latinx. In unadjusted analyses, support for AP and OTC (AP: 48.9% before; 95% CI, 47.1% to 50.6%; to 55.1% after; 95% CI, 52.8% to 57.3%; *P* < .001; OTC: 49.4% before; 95% CI, 47.6% to 51.1%; to 55.2% after; 95% CI, 52.9% to 57.5%; *P* < .001) and personal interest in AP and OTC (AP: 23.6% before; 95% CI, 22.2% to 25.1%; to 26.4% after; 95% CI, 24.3% to 28.4%; *P* = .02; OTC: 36.0% before; 95% CI, 34.3% to 37.6%; to 42.5% after; 95% CI, 40.2% to 44.7%; *P* < .001) increased significantly by year (Table 2 and Table 3). In adjusted analyses, support for AP (50.0% before; 95% CI, 48.5% to 51.5%; to 54.0% after; 95% CI, 52.0% to 55.9%) and OTC (50.4% before; 95% CI, 48.9% to 51.9%; to 54.3% after; 95% CI, 52.4% to 56.2%) and personal interest in OTC (36.9% before; 95% CI, 35.4% to 38.4;% to 41.5% after; 95% CI, 39.4% to 43.5%) increased significantly by year (Table 3); personal interest in AP did not. See eTable in Supplement 1 for full multivariable results.

**Table 1.** Participant Demographic Characteristics and Health Care Experiences by Survey Year (N = 10 543)

Participant demographic characteristics and health care experiences	Participants, No. (weighted %)		*P* value^a^
	Before *Dobbs, *2021-2022 (n = 6982)	After *Dobbs, *2023 (n = 3561)	
Race			
Asian or Pacific Islander (non-Hispanic)	247 (5.7)	132 (5.1)	.41
Black (non-Hispanic)	594 (13.7)	304 (13.6)	.99
White (non-Hispanic)	4504 (54.6)	2270 (54.2)	[Reference]
>1 race or other race^b^	242 (4.6)	147 (5.8)	.10
Ethnicity and language			
Hispanic/Latinx, completed English survey	947 (14.8)	471 (15.3)	.61
Hispanic/Latinx, completed Spanish survey	448 (6.5)	237 (6.1)	.62
Age group, y			
15-17	175 (9.0)	196 (8.8)	.74
18-19	60 (2.6)	56 (6.1)	<.001
20-24	375 (13.0)	207 (13.0)	.56
25-29	1022 (15.6)	424 (14.5)	[Reference]
30-39	2666 (31.3)	1258 (30.1)	.71
40-49	2684 (28.5)	1420 (27.4)	.70
LGBTQI+			
Yes	902 (13.9)	585 (19.5)	<.001
Missing	3 (<0.1)	9 (<0.1)	NA
FPL			
<100% FPL	1115 (12.2)	516 (10.3)	[Reference]
100%-199% FPL	1114 (14.1)	514 (12.7)	.57
≥200% FPL	4753 (73.7)	2531 (76.9)	.01
MSA			
Non-MSA	906 (11.7)	495 (12.0)	[Reference]
MSA	6076 (88.3)	3066 (88.0)	.69
Geographic region			
Northeast	1083 (16.6)	540 (16.8)	[Reference]
Midwest	1762 (20.2)	910 (20.3)	.93
South	2496 (38.7)	1260 (38.8)	.92
West	1641 (24.5)	851 (24.2)	.78
Living in a state with a total or near abortion ban in 2023^c^			
>18 wk legal	4284 (60.3)	2139 (57.7)	[Reference]
6- to 18-wk ban	1172 (17.1)	606 (19.2)	.03
Total ban	1526 (22.6)	816 (23.1)	.29
Political party			
Republican	1502 (20.9)	738 (20.6)	.11
Democrat	2875 (42.5)	1391 (40.8)	.02
Independent	1685 (23.6)	926 (26.5)	[Reference]
Something else	908 (13.1)	450 (12.1)	.05
Missing	12 (<0.1)	56 (<0.1)	NA
Religion			
None/Atheist/Agnostic	2092 (30.4)	1154 (33.9)	[Reference]
Catholic	1503 (22.5)	687 (19.9)	<.001
Evangelical/Protestant	2013 (28.7)	1003 (27.5)	.03
Mormon	140 (1.8)	73 (2.0)	.96
Jewish	117 (1.6)	62 (1.6)	.74
Other Christian religion	813 (10.6)	373 (10.6)	.26
Other non-Christian religion	288 (4.5)	159 (4.4)	.42
Missing	16 (<0.1)	50 (<0.1)	NA
Pregnancy and abortion history			
Nulliparous	2705 (46.1)	1413 (48.8)	[Reference]
History of pregnancy, no abortion	3449 (43.1)	1726 (41.1)	.06
History of procedural abortion but no medication abortion	564 (7.4)	268 (6.4)	.04
History of medication abortion	248 (3.4)	148 (3.6)	.85
Missing	16 (<0.1)	6 (<0.1)	NA
Health care experiences			
No. of barriers trying to access RH services in past 3 y			
None	3238 (42.6)	1477 (38.8)	[Reference]
1	975 (11.7)	533 (13.4)	.007
2	640 (8.3)	326 (8.4)	.28
≥3	1184 (15.1)	699 (17.9)	.001
Never tried	931 (22.1)	518 (21.4)	.46
Missing	14 (<0.1)	8 (<0.1)	NA
Ever experienced medical mistreatment			
None	4014 (62.3)	1990 (58.5)	[Reference]
Made to feel symptoms were not real, severe, or important (only, no ridicule or humiliation)	1269 (17.1)	669 (18.5)	.06
Ridiculed or humiliated (may have also experienced neglect)	1683 (20.6)	894 (23.0)	.01
Missing	16 (<0.1)	8 (<0.1)	NA

Abbreviations: *Dobbs, Dobbs v Jackson Women’s Health*; FPL, federal poverty level; LGBTQI+, lesbian, gay, bisexual, transgender, queer, intersex, or other; MSA, metropolitan statistical area; NA, not applicable; RH, reproductive health.

^a^ Based on weighted bivariable logistic regression analyses using robust SEs to account for multiple observations per individual.

^b^ Category included people who identified as American Indian or Alaska Native (36 participants), people who selected more than one race (352 participants), and 1 person who selected other.

^c^ Total abortion ban states included Alabama, Arkansas, Louisiana, Indiana, Kentucky, Missouri, Mississippi, North Dakota, Oklahoma, South Dakota, Tennessee, and Texas; 6- to 18-week ban states included Arizona, Florida, Georgia, North Carolina, Nebraska, South Carolina, Utah, and West Virginia.

**Table 2.** Support for and Personal Interest in Advance Provision and Over-the-Counter (OTC) Access of Medication Abortion by Survey Year

Participant responses	No. (weighted column %**)**^a^		*P* value^a^
	Before *Dobbs*, 2021-2022 (n = 6982)	After *Dobbs*, 2023 (n = 3561)	
Support for advance provision (somewhat/strongly in favor)	3497 (48.9)	1947 (55.1)	<.001
Strongly in favor	2176 (30.2)	1336 (37.8)	
Somewhat in favor	1321 (18.7)	611 (17.3)	
Somewhat opposed	746 (10.8)	362 (10.5)	
Strongly opposed	1722 (24.5)	820 (21.8)	
Not sure	933 (15.9)	419 (12.6)	
Missing	24 (<0.1)	13 (<0.1)	
Personal interest in advance provision (definitely/probably yes)	1622 (23.6)	892 (26.4)	.02
Definitely yes	749 (10.6)	402 (11.3)	
Probably yes	873 (13.1)	490 (15.1)	
Probably no	1109 (15.7)	583 (16.2)	
Definitely no	3462 (48.5)	1735 (46.4)	
I don’t know	780 (12.2)	346 (10.9)	
Missing	9 (<0.1)	5 (<0.1)	
Support for OTC access (somewhat/strongly in favor)	3502 (49.4)	1926 (55.2)	<.001
Strongly in favor	2238 (30.4)	1283 (36.0)	
Somewhat in favor	1264 (18.9)	643 (19.2)	
Somewhat opposed	727 (10.5)	352 (9.6)	
Strongly opposed	1789 (25.0)	862 (23.4)	
Not sure	920 (15.1)	388 (11.9)	
Missing	44 (<0.1)	33 (<0.1)	
Personal interest in OTC access (definitely/probably yes)	2548 (36.0)	1496 (42.5)	<.001
Definitely yes	1422 (19.9)	916 (25.7)	
Probably yes	1126 (16.0)	580 (16.8)	
Probably no	704 (10.6)	362 (10.4)	
Definitely no	2917 (41.1)	1323 (35.5)	
I don’t know	774 (12.4)	361 (11.6)	
Missing	39 (<0.1)	19 (<0.1)	

Abbreviation: *Dobbs, Dobbs v Jackson Women’s Health*.

^a^ All analyses included raw frequences and weighted percentages.

**Table 3.** Changes in Personal Interest in and Support for Advance Provision and Over-the-Counter (OTC) Access of Medication Abortion From Before to After *Dobbs v Jackson Women’s Health* (*Dobbs*) According to Bivariable and Multivariable Logistic Regression Analyses

Survey year	Advance provision				OTC access			
	Support		Personal interest		Support		Personal interest	
	% (95% CI)	OR (95% CI)	% (95% CI)	OR(95% CI)	% (95% CI)	OR (95% CI)	% (95% CI)	OR (95% CI)
Unadjusted analyses, No.	10 506		10 354		10 466		10 485	
Before *Dobbs*, 2021-2022	48.9 (47.1 to 50.6)	1 [Reference]	23.6 (22.2 to 25.1)	NA	49.4 (47.6 to 51.1)	1 [Reference]	36.0 (34.3 to 37.6)	1 [Reference]
After *Dobbs*, 2023	55.1 (52.8 to 57.3)	1.28 (1.15 to 1.43)	26.4 (24.3 to 28.4)	1.16 (1.02 to 1.32)	55.2 (52.9 to .57.5)	1.26 (1.13 to 1.41)	42.5 (40.2 to 44.7)	1.32 (1.18 to 1.47)
Unadjusted difference	6.2 (3.5 to 8.9)	NA	2.8 (3.5 to 5.2)	NA	5.8 (3.1 to 8.5)	NA	6.5 (3.9 to 9.2)	NA
Adjusted analyses, No.^a^	10 336		10 354		10 299		10 314	
Before *Dobbs*, 2021-2022	50.0 (48.5 to 51.5)	1 [Reference]	24.4 (23.0 to 25.7)	NA	50.4 (48.9 to 51.9)	1 [Reference]	36.9 (35.4 to 38.4)	NA
After *Dobbs*, 2023	54.0 (52.0 to 55.9)	1.24 (1.09 to 1.41)	25.4 (23.5 to 27.3)	1.07 (0.93 to 1.24)	54.3 (52.4 to 56.2)	1.23 (1.08 to 1.40)	41.5 (39.4 to 43.5)	1.27 (1.12 to 1.45)
Adjusted difference^a^	4.0 (1.6 to 6.4)	NA	1.1 (−1.2 to 3.3)	NA	3.9 (1.5 to 6.3)	NA	4.6 (2.1 to 7.0)	NA

Abbreviations: NA, not applicable; OR, odds ratio.

^a^ Analyses adjusted for race, ethnicity, and survey language; age group; lesbian, gay, bisexual, transgender, queer, intersex, plus identity; poverty level; geographic region; living in a metropolitan statistical area; living in state that bans abortion; religion; political party affiliation; pregnancy history; experienced barriers trying to access reproductive health care services; and experience of medical mistreatment. Analyses are based on weighted logistic regression accounting for multiple observations per individual and include marginal estimated values.

The selected advantages and disadvantages aggregated across survey years are presented in Table 4. The most common advantages selected for AP and OTC were privacy (5655 respondents [53.3%] and 4739 respondents [44.3%], respectively), convenience (5160 respondents [48.8%] and 5439 respondents [51.7%], respectively), and potential to access abortion earlier in pregnancy (5167 respondents [48.4%] and 5044 respondents [46.6%], respectively). The most common disadvantages selected for AP and OTC included that people might take the pills incorrectly (6059 respondents [57.2%] and 5656 respondents [53.7%], respectively), people may have unanswered questions before the abortion (4858 respondents [46.3%] and 4939 respondents [47.1%], respectively), and concerns that someone might be forced to take the pills without their consent (4930 respondents [46.7%] and 4641 respondents [43.9%], respectively).

**Table 4.** Advantages and Disadvantages of Alternative Forms of Medication Abortion Provision Before (2021-2022) and After (2023) *Dobbs v Jackson Women’s Health*^a^

Advantages and disadvantages	No. (weighted %**)**	
	Advance provision	OTC access
Advantages		
Could help get the abortion earlier in pregnancy	5167 (48.4)	5044 (46.6)
Could be more convenient	5160 (48.8)	5439 (51.7)
Could be more private	5655 (53.3)	4739 (44.3)
Could be less expensive	3755 (35.0)	4264 (40.1)
Could avoid going to a clinic	4165 (39.3)	4614 (43.9)
Could avoid having to see a doctor or nurse	3206 (30.2)	4033(38.6)
Could be safer	2843 (25.8)	1843 (16.6)
Could be more effective	2680 (25.1)	1887 (17.1)
Other	219 (1.9)	163 (1.3)
I don’t see any advantages	2884 (28.2)	2807 (27.9)
Disadvantages		
People might take the pills incorrectly	6059 (57.2)	5656 (53.7)
People’s questions may not be answered before the abortion	4858 (46.3)	4939 (47.1)
Someone might be forced to take the pills without their consent	4930 (46.7)	4641 (43.9)
People might not see a doctor or nurse before the abortion	3936 (37.2)	4207 (40.1)
Could be less safe	3776 (35.6)	3852 (36.3)
Could be too convenient	3113 (27.4)	3209 (29.4)
Could be less effective	2101 (19.9)	2076 (19.3)
Could be more expensive	1386 (12.9)	1427 (13.8)
Could be less private	514 (4.9)	1164 (11.0)
Other	551 (4.8)	448(3.9)
I don’t see any disadvantages	1306 (14.6)	1490 (16.0)

Abbreviation: OTC, over-the-counter.

^a^ Number of respondents ranges from 10 290 to 10 396.

In unadjusted analyses, population groups with significant increases in personal interest in AP included being non-Hispanic White race (4.6 percentage points [pp]; 95% CI, 1.5 to 7.7 pp), living in the Midwest (5.1 pp; 95% CI, 0.1 to 10.1 pp) or in a state with a total abortion ban (5.3 pp; 95% CI, 0.5 to 10.3 pp vs people in states without bans 1.4 pp; 95% CI, −1.7 to 4.6 pp), Democratic political party affiliation (7.0 pp; 95% CI, 2.8 to 11.1 pp), other Christian religion (9.2 pp; 95% CI, 1.9 to 16.4 pp), had never been pregnant (4.8 pp; 95% CI, 0.6 to 8.9 pp), and had experienced ridicule or humiliation from a health care practitioner (7.0 pp; 95% CI, 1.5 to 12.3 pp) (Table 5). People with a history of procedural abortion but no medication abortion were the only group to report a significant decline in personal interest in AP (−9.9 pp; 95% CI, −18.1 to −1.7 pp). Most population groups reported significant increases in personal interest in OTC (Table 5) and included people who identified as Hispanic/Latinx ethnicity and completed the survey in English (8.5 pp; 95% CI, 1.2 to 15.9 pp), non-Hispanic White race (7.4 pp; 95% CI, 4.1 to 10.8 pp), those aged 15 to 17 years (16.8 pp; 95% CI, 5.6 to 28.1 pp), those aged 30 to 39 years (4.1 pp; 95% CI, 0.2 to 8.1 pp), those aged 40 to 49 years (6.1 pp; 95% CI, 2.3 to 9.8 pp), Democratic political affiliation (11.9 pp; 95% CI, 7.7 to 16.2 pp), those with history of medication abortion (16.2 pp; 95% CI, 4.9 to 27.4 pp), those living at less than 100% of the FPL (9.3 pp; 95% CI, 1.9 to 16.8 pp) or 200% or greater than the FPL (5.9 pp; 95% CI, 2.8 to 9.0 pp), those in a state with a total abortion ban (9.4 pp; 95% CI, 3.9 to 14.9 pp vs people in states without bans, 5.4 pp; 95% CI, 2.0 to 8.9 pp), those in every geographic region except the Northeast, and those who recently experienced 3 or more barriers (9.8 pp; 95% CI, 3.4 to 16.3 pp) or never tried (8.7 pp; 95% CI, 1.9 to 15.6 pp) to access RH care (Table 5).

**Table 5.** Changes in Personal Interest in Advance Provision and Over-the-Counter (OTC) Access of Medication Abortion From 2021-2022 (N = 6982) to 2023 (N = 3561), by Participant Characteristics and Health Care Experiences

Participant demographic characteristics and health care experiences	Interest in advance provision				Interest in OTC			
	Weighted row %			*P* value	Weighted row %			*P* value
	2021-2022	2023	Change (95% CI)^a^		2021-2022	2023	Change (95% CI)^a^	
Total	23.6	26.4	2.8 (3.5 to 5.2)	.02	36.0	42.5	6.5 (3.9 to 9.2)	<.001
Race								
Asian or Pacific Islander (non-Hispanic)	27.0	27.7	0.7 (−9.9 to 11.3)	.90	43.0	46.9	3.9 (5.6 to 28.1)	.54
Black (non-Hispanic)	24.2	25.5	1.2 (−6.0 to 8.5)	.74	36.3	42.6	6.3 (−2.1 to 14.7)	.14
More than 1 race or other race^b^	31.3	26.8	−4.5 (−1.6 to 7.4)	.46	44.3	42.9	−1.4 (−14.6 to 11.9)	.84
White (non-Hispanic)	21.6	26.3	4.6 (1.5 to 7.7)	<.001	35.6	43.0	7.4 (4.1 to 10.8)	<.001
Ethnicity and language								
Hispanic/Latinx completed English survey	30.7	34.1	3.4 (−3.8 to 10.6)	.36	39.9	48.4	8.5 (1.2 to 15.9)	.02
Hispanic/Latinx completed Spanish survey	14.6	9.4	−5.3 (−12.1 to 1.5)	.13	17.2	18.6	1.4 (−6.7 to 9.5)	.74
Age group, y								
15-17	21.1	23.6	2.5 (−7.6 to 12.6)	.63	24.2	41.0	16.8 (5.6 to 28.1)	.003
18-19	23.1	34.5	11.4 (−2.0 to 18.8)	.20	44.9	50.9	6.0 (−13.3 to 25.3)	.54
20-24	29.5	38.8	9.3 (−2.0 to 1.9)	.06	43.9	48.6	4.7 (−5.1 to 14.5)	.34
25-29	29.2	31.0	1.8 (−4.6 to 8.2)	.58	41.8	47.5	5.7 (−1.1 to 12.5)	.10
30-39	22.7	25.4	2.7 (−0.7 to 6.1)	.11	36.8	40.9	4.1 (0.2 to 8.1)	.04
40-49	19.8	18.3	−1.5 (−4.6 to 1.6)	.34	31.2	37.3	6.1 (2.3 to 9.8)	.002
LGBTQI+ identity								
Yes	39.5	40.3	0.9 (−0.6 to 4.4)	.13	55.5	57.5	2.0 (−5.4 to 9.3)	.60
No	21.1	23.1	1.9 (−6.3 to 8.1)	.81	32.8	38.9	6.0 (3.2 to 8.8)	<.001
FPL								
<100% FPL	19.2	24.2	5.0 (−1.6 to 11.6)	.14	27.5	36.8	9.3 (1.9 to 16.8)	.01
100%-199% FPL	17.1	21.2	4.1 (−2.0 to 10.0)	.19	26.4	31.4	5.0 (−1.7 to 11.8)	.15
≥200% FPL	25.6	27.6	2.0 (−0.9 to 4.9)	.18	39.2	45.1	5.9 (2.8 to 9.0)	<.001
MSA								
MSA	25.0	27.4	2.4 (−0.2 to 5.1)	.07	37.7	43.9	6.2 (3.3 to 9.0)	<.001
Non-MSA	13.4	19.1	5.7 (−0.3 to 11.8)	.06	22.8	32.2	9.4 (2.4 to 16.4)	.01
Geographic region								
Northeast	26.2	24.4	−1.8 (−7.4 to 3.8)	.53	42.8	41.8	−1.0 (−7.3 to 5.4)	.77
Midwest	19.9	25.0	5.1 (0.1 to 10.1)	.04	33.3	42.8	9.5 (3.8 to 15.0)	.001
South	23.2	26.9	3.7 (−0.4 to 7.8)	.07	33.3	41.6	8.3 (3.9 to 12.8)	<.001
West	25.7	28.2	2.5 (−2.5 to 7.5)	.32	37.7	44.1	6.4 (1.0 to 11.8)	.02
Lives in a state with total or near abortion ban in 2023^c^								
>18 wk is legal	25.5	26.9	1.4 (−1.7 to 4.6)	.36	39.6	45.0	5.4 (2.0 to 8.9)	.002
6 to 18-wk ban	23.1	27.2	4.1 (−2.1 to 10.3)	.20	34.5	41.9	7.4 (0.9 to 14.0)	.03
Total ban	19.2	24.5	5.3 (0.5 to 10.3)	.03	27.3	36.7	9.4 (3.9 to 14.9)	.001
Political party								
Republican	9.2	11.0	1.8 (2.7 to 9.2)	.40	16.0	20.6	4.6 (−0.3 to 9.4)	.07
Democrat	32.9	39.9	7.0 (2.8 to 11.1)	<.001	48.5	60.4	11.9 (7.7 to 16.2)	<.001
Independent	21.9	21.6	−0.4 (−4.9 to 4.2)	.87	34.0	39.1	5.0 (−0.2 to 10.3)	.06
Something else	20.2	19.9	−0.3 (−7.5 to 6.8)	.93	30.7	30.7	−0.0 (−7.6 to 7.5)	.99
Religion								
None/Atheist/Agnostic	37.8	37.9	0.1 (−4.7 to 4.8)	.98	54.3	58.8	4.5 (−0.3 to 9.5)	.07
Catholic	20.7	21.7	1.0 (−4.4 to 6.3)	.73	30.6	38.1	7.5 (1.6 to 13.4)	.01
Evangelical or protestant	13.2	16.8	3.6 (−0.3 to 7.5)	.07	23.4	29.1	5.7 (1.0 to 10.3)	.02
Mormon	2.5	5.5	3.0 (−6.9 to 12.9)	.56	7.0	10.4	3.4 (−6.9 to 13.8)	.52
Jewish	29.1	23.9	−5.2 (−19.8 to 9.4)	.48	52.5	53.6	1.1 (−17.7 to 19.8)	.91
Other Christian religion	15.5	24.7	9.2 (1.9 to 16.4)	.01	26.1	32.7	6.6 (−1.2 to 14.5)	.10
Other non-Christian religion	33.3	32.9	−0.4 (−12.4 to 11.6)	.95	48.7	54.9	6.2 (−7.0 to 19.5)	.36
History of pregnancy and abortion								
Never pregnant	28.8	33.6	4.8 (0.6 to 8.9)	.03	41.5	49.4	7.9 (3.5 to 12.3)	<.001
History of pregnancy, no abortion	13.2	15.0	1.8 (−0.8 to 4.4)	.17	25.2	30.9	5.8 (2.5 to 9.0)	<.001
History of procedural abortion but no medication abortion	38.8	28.9	−9.9 (−18.1 to −1.7)	.02	52.9	44.5	−8.4 (−17.6 to 0.8)	.07
History of medication abortion	53.8	55.6	1.8 (−11.6 to 15.2)	.79	61.9	78.1	16.2 (4.9 to 27.4)	.01
No. of barriers trying to access RH services in past 3 y								
None	20.2	20.6	0.4 (−2.9 to 3.6)	.83	33.5	39.2	5.7 (1.9 to 9.6)	.003
1	29.8	30.2	0.4 (−6.5 to 7.3)	.91	44.3	45.3	1.0 (−6.3 to 8.3)	.79
2	30.3	38.0	7.7 (−1.7 to 17.0)	.11	50.4	50.5	0.1 (−9.2 to 9.5)	.98
3-10	30.7	34.4	3.7 (−2.3 to 9.7)	.23	42.4	52.0	9.8 (3.4 to 16.3)	.003
Never tried to access RH services in past 3 y	19.8	23.0	3.2 (−3.1 to 9.5)	.32	26.8	35.5	8.7 (1.9 to 15.6)	.01
Ever experienced any medical mistreatment								
None	19.8	20.9	1.1 (−1.9 to 4.2)	.46	30.1	36.6	6.5 (3.2 to 10.0)	<.001
Neglect only (made to feel symptoms ignored/not important)	29.7	30.1	3.9 (−5.7 to 6.5)	.89	43.8	45.8	4.8 (2.7 to 9.2)	.55
Ridiculed or humiliated, could include neglect	30.6	37.5	7.0 (1.5 to 12.3)	.01	47.8	55.0	7.3 (1.8 to 12.9)	.01

Abbreviations: FPL, federal poverty level; LGBTQI+, lesbian, gay, bisexual, transgender, queer, intersex, or other; MSA, metropolitan statistical area; RH, reproductive health.

^a^ Differences are based on weighted logistic regression analyses using group by time interactions and marginal contrasts accounting for clustering of multiple observations by individual; all percentages are weighted.

^b^ This category included people who identified as American Indian or Alaska Native (36 participants), people who selected more than one race (352 participants), and 1 person who selected other.

^c^ Total abortion ban states included Alabama, Arkansas, Louisiana, Indiana, Kentucky, Missouri, Mississippi, North Dakota, Oklahoma, South Dakota, Tennessee, and Texas; 6- to 18-week ban states included Arizona, Florida, Georgia, North Carolina, Nebraska, South Carolina, Utah, and West Virginia.

## Discussion

We found significant increases in national support for and personal interest in AP and OTC access to medication abortion across a wide range of population groups, from just before and after the removal of federal protections on abortion. Both years of data reveal much greater national levels of support for and interest in AP and OTC access than previously reported.^22^ By mid-2023, over half of the general public was supportive of these less medicalized models of abortion care. Notably, personal interest in the OTC model was much higher and increases were much steeper when compared with interest in the AP model. The OTC model was particularly appealing among people experiencing marginalization from the health care system, including people living in states with total abortion bans, in nonurban areas, living in poverty, and those who experienced barriers to reproductive health care and medical mistreatment. Unlike the AP model, the OTC model may be particularly appealing for people who lack access to high-quality, nonjudgmental, and respectful health care because it does not require interaction with a clinician.

The general public’s growing interest in alternative models of abortion care may be explained by increasing awareness and use of medication abortion,^14,29^ as well as extraordinary shifts in the abortion policy landscape,^28^ given that people from abortion-ban states experienced more dramatic increases in personal interest in both AP and OTC access compared with their counterparts. The rapid growth in patients accessing abortion via telehealth and online models of care^3,12,30^ since the FDA lifted the in-person dispensing requirement for mifepristone may also have increased people’s comfort with and trust in models of care that allow people to self-assess for eligibility, monitor symptoms, and self-manage their abortions. Additionally, the extensive coverage of the US Supreme Court case *Alliance for Hippocratic Medicine v. US Food and Drug Administration* may have helped to inform the general public about the safety of medication abortion and increased concerns about impediments for future access.^31,32,33,34^ If facility-based abortion care in the US continues to become less accessible, we may continue to observe increasing support for less medicalized, more person-focused participatory models of abortion care.

Consistent with previous research,^18,22^ support for these models of care varied significantly by ideological perspectives, including political party affiliation and religion, with Catholics, Evangelicals, and Protestants reporting significantly lower yet increasing interest in an OTC model when compared with people reporting no religion. Health history was also associated with people’s views about OTC and AP access. People with a history of medication abortion reported some of the highest levels of personal interest in both the AP and OTC models, although people with a history of procedural abortion but no medication abortion were the only group to report a decline in interest in AP, reminding us of the importance of offering people a range of abortion care options.

Young people aged less than 18 years are disproportionately impacted by abortion bans due to parental involvement laws and greater financial and logistical constraints on travel,^35^ and they reported the largest increase in personal interest in OTC access across population subgroups (16.8 pp). This finding highlights how young people may be particularly interested in more autonomous and private models of abortion care.^35^

Similar to previous work,^18,22,23^ about half of people noted that both models offer the benefits of earlier access to abortion, convenience, and privacy. However, participants also noted that people might take the pills incorrectly, have questions left unanswered, or be forced to take the pills without consent, factors that will require consideration if these models are broadly implemented.

While it is unlikely that states with abortion bans would provide medication abortion in advance or sell it OTC at retail pharmacies should the FDA approve it, these models may still improve abortion access for people living in such states. By expanding access to abortion to brick-and-mortar and/or online pharmacies and via telehealth in states with protected access to abortion, these alternatives would offer more options where people could travel to obtain AP or OTC medication abortion or receive the medications from friends or family members living in states with such access. Furthermore, people living in states with abortion bans could receive the medications in advance under shield laws, which add legal protections for clinicians in some protected access states who provide telehealth services to patients in states with abortion bans or restrictions.^36,37^ In an analysis^15^ of over 48 000 AP requests, demand for AP increased after *Dobbs*, particularly among people living in states that anticipated abortion bans.

### Limitations

Our study has some limitations. While the timing of data collection—just a few months before and 1 year after *Dobbs—*allowed us to capture changes in people’s views that could have resulted from this unprecedented policy shift, our serial cross-sectional design precludes us from attributing these changes to *Dobbs* as they may be explained by other unmeasured factors. Furthermore, while an important study strength is that our outcome items were drawn from the literature and further refined through cognitive testing, these questions may have been difficult for people to interpret, in particular for people with limited knowledge about abortion and for people living in states with abortion bans, who may not have understood whether these models could offer any benefit to them personally. Additionally, our findings only offer a perceived indication of whether people would utilize these models of care within the real-world context.

## Conclusions

In this serial cross-sectional national study of AFAB people aged 15 to 49 years, we found that national support for and interest in expanded access to medication abortion has increased, particularly for people living in restrictive settings and those marginalized by the health care system. These findings suggest that AP and OTC access to medication abortion offer 2 promising approaches to abortion care. Future research should further examine the safety, acceptability, appropriateness, adoption, and feasibility of implementing these models of care. Given the high level of interest and support for AP and OTC access, policymakers and regulators should support and facilitate the research needed for the approval of an OTC product, as well as prioritize policies that will ensure that both AP and OTC products are covered by insurance so that these approaches can become broadly available to the general public.

## Notes

Supplement 1. **eFigure. **Survey Language Used to Describe Advance Provision and OTC Access to Medication Abortion **eTable. **Multivariate Associations Between Participant Characteristics and Adjusted Odds (aOR) of Support for and Personal Interest in Advance Provision (AP) and Over-the-Counter (OTC) Access of Medication Abortion Before *Dobbs *and After *Dobbs*

Supplement 2.
Data Sharing Statement
