# Supplementary material for: Changes in Support for Advance Provision and Over-the-Counter Access to Medication Abortion
Source: JAMA Netw Open. 2025 Jan 16;8(1):e2454767. doi: 10.1001/jamanetworkopen.2024.54767 (PMC11739987; doi:10.1001/jamanetworkopen.2024.54767)
Supplement: Supplement 1. — eFigure. Survey Language Used to Describe Advance Provision and OTC Access to Medication Abortion eTable. Multivariate Associations Between Participant Characteristics and Adjusted Odds (aOR) of Support for and Personal Interest in Advance Provision (AP) and Over-the-Counter (OTC) Access of Medication Abortion Before Dobbs and After Dobbs [file jamanetwopen-e2454767-s001.pdf]

Supplemental Online Content

Biggs MA, Schroeder R, Kaller S, Grossman D, Scott KA, Ralph LJ. Changes in support for advance provision and over-the-counter access to medication abortion. *JAMA Netw Open*. 2025;8(1):e2454767. doi:10.1001/jamanetworkopen.2024.54767

eFigure. Survey Language Used to Describe Advance Provision and OTC Access to Medication Abortion

eTable. Multivariate Associations Between Participant Characteristics and Adjusted Odds (aOR) of Support for and Personal Interest in Advance Provision (AP) and Over-the-Counter (OTC) Access of Medication Abortion Before *Dobbs* and After *Dobbs*

This supplemental material has been provided by the authors to give readers additional information about their work.

© 2025 Briggs MA et al. *JAMA Network Open*.

eFigure. Survey Language Used to Describe Advance Provision and OTC Access to Medication Abortion

Medication abortion, or the abortion pill, is a safe and effective way to end a pregnancy. It involves taking two types of pills, about 1 day apart. Currently, people usually need to go in person to a doctor's office or clinic to get the pills or sometimes they can get the pills from a doctor in the mail. In the future, people may also be able to get abortion pills over the counter or in advance from a doctor.

On the next page you will see some new ideas about ways to get abortion pills. When answering the following questions, don't focus on cost because it is too early to know the costs of each option.

Advance Provision: Getting abortion pills ahead of time from a doctor just in case

You would get abortion pills from a doctor ahead of time, just in case. Later, if you got pregnant, you could use the pills at home if you needed an abortion. You would get written information about how to use the pills.

Over-the-Counter (OTC): Getting abortion pills over the counter from a pharmacy without a prescription

If you needed an abortion, you could get abortion pills without a prescription over the counter in a pharmacy, drugstore or grocery store, just like aspirin, condoms or pregnancy tests.

© 2025 Briggs MA et al. *JAMA Network Open*.

eTable. Multivariate Associations Between Participant Characteristics and Adjusted Odds (aOR) of Support for and Personal Interest in Advance Provision (AP) and Over-the-Counter (OTC) Access of Medication Abortion Before *Dobbs* and After *Dobbs*

	Advance Provision				OTC			
	Support (n=10,336)		Personal interest (n=10,354)		Support (n=10,299)		Personal interest (n=10,314)	
**Participant demographic characteristics and health care experiences**	**aOR**	**(95% CI)**	**aOR**	**(95% CI)**	**aOR**	**(95% CI)**	**aOR**	**(95% CI)**
Survey year								
Pre-Dobbs, 2021-22	Ref.				Ref.			
Post-Dobbs, 2023	1.24	(1.09,1.41)	1.07	(0.92,1.24)	1.23	(1.08,1.40)	1.27	(1.12,1.45)
Race/ethnicity and language								
Asian or Pacific Islander (non-Hispanic)	0.68	(0.47,0.97)	0.94	(0.65,1.36)	0.60	(0.43,0.83)	0.91	(0.65,1.28)
Black (non-Hispanic)	0.64	(0.50,0.80)	0.90	(0.69,1.17)	0.56	(0.44,0.70)	0.79	(0.63,1.00)
Hispanic/Latinx completed English survey	0.90	(0.73,1.11)	1.37	(1.09,1.71)	0.86	(0.69,1.06)	1.08	(0.87,1.34)
Hispanic/Latinx completed Spanish survey	0.52	(0.36,0.73)	0.70	(0.47,1.04)	0.43	(0.30,0.61)	0.45	(0.31,0.64)
More than one race or other race[a]	0.89	(0.65,1.23)	1.19	(0.85,1.66)	0.81	(0.59,1.13)	1.01	(0.73,1.38)
White (non-Hispanic)	Ref.				Ref.			
Age group								
15-17	1.09	(0.75,1.59)	0.96	(0.65,1.43)	0.85	(0.58,1.24)	0.89	(0.61,1.29)
18-19	1.32	(0.79,2.21)	1.06	(0.63,1.81)	1.29	(0.80,2.09)	1.6	(0.98,2.61)
20-24	1.20	(0.90,1.59)	1.10	(0.83,1.45)	1.12	(0.84,1.49)	1.13	(0.85,1.49)
25-29	Ref.				Ref.			
30-39	0.95	(0.78,1.16)	0.83	(0.67,1.02)	0.94	(0.77,1.16)	0.87	(0.72,1.07)
40-49	1.07	(0.87,1.32)	0.82	(0.65,1.03)	0.91	(0.74,1.13)	0.89	(0.72,1.10)
LGBTQ+ identity	1.96	(1.57,2.46)	1.31	(1.07,1.62)	1.58	(1.27,1.97)	1.38	(1.12,1.70)
Federal Poverty Level (FPL), <100% FPL	Ref.				Ref.			
100-199% FPL	1.01	(0.77,1.32)	0.91	(0.68,1.23)	1.10	(0.84,1.45)	0.91	(0.69,1.19)
>=200% FPL	1.67	(1.34,2.09)	1.35	(1.05,1.73)	1.80	(1.42,2.28)	1.47	(1.16,1.86)
Metropolitan statistical area (MSA) (vs non-MSA)	1.35	(1.11,1.65)	1.33	(1.03,1.72)	1.50	(1.23,1.84)	1.35	(1.09,1.68)
Geographic region, Northeast	Ref.				Ref.			
Midwest	1.11	(0.89,1.38)	0.93	(0.73,1.19)	1.11	(0.89,1.39)	0.96	(0.77,1.20)
South	1.41	(1.09,1.81)	1.22	(0.92,1.61)	1.44	(1.11,1.86)	1.17	(0.91,1.51)
West	1.03	(0.83,1.30)	1.04	(0.82,1.32)	1.12	(0.89,1.40)	1.00	(0.81,1.25)
Lives in state with total/near abortion ban in 2023[b]								
> 18 weeks is legal	Ref.				Ref.			
6 to 18-week ban	0.96	(0.76,1.20)	0.98	(0.76,1.26)	0.86	(0.68,1.08)	0.94	(0.75,1.18)
Total ban	0.87	(0.70,1.07)	0.95	(0.74,1.22)	0.83	(0.67,1.03)	0.79	(0.63,0.99)

eTable. Multivariate Associations Between Participant Characteristics and Adjusted Odds (aOR) of Support for and Personal Interest in Advance Provision (AP) and Over-the-Counter (OTC) Access of Medication Abortion Pre-Dobbs and Post-Dobbs *(continued)*

(continued)	**Advance Provision**				**OTC**			
	**Support (n=10,336)**		**Personal interest (n=10,354)**		**Support (n=10,299)**		**Personal interest (n=10,314**	
**Participant demographic characteristics and health care experiences**	**aOR**	**(95% CI)**	**aOR**	**(95% CI)**	**aOR**	**(95% CI)**	**aOR**	**(95% CI)**
Religion, None/Atheist/Agnostic	Ref.				Ref.			
Catholic	0.47	(0.38,0.57)	0.59	(0.47,0.72)	0.49	(0.40,0.60)	0.54	(0.45,0.66)
Evangelical or protestant	0.30	(0.25,0.36)	0.39	(0.32,0.48)	0.32	(0.26,0.38)	0.38	(0.32,0.45)
Mormon	0.15	(0.09,0.26)	0.11	(0.03,0.33)	0.16	(0.09,0.29)	0.12	(0.05,0.28)
Jewish	0.33	(0.22,0.51)	0.62	(0.38,1.02)	0.51	(0.32,0.79)	0.87	(0.55,1.36)
Other Christian religion	0.35	(0.28,0.44)	0.47	(0.37,0.61)	0.37	(0.30,0.47)	0.39	(0.31,0.49)
Other non-Christian religion	0.91	(0.64,1.30)	0.95	(0.67,1.36)	0.92	(0.65,1.31)	0.92	(0.66,1.28)
Political party								
Republican	0.49	(0.40,0.61)	0.59	(0.44,0.78)	0.47	(0.39,0.58)	0.51	(0.41,0.64)
Democrat	2.52	(2.13,2.99)	1.83	(1.53,2.19)	2.37	(2.01,2.81)	1.85	(1.58,2.18)
Independent	Ref.				Ref.			
Something else	0.79	(0.64,0.99)	0.85	(0.65,1.12)	0.80	(0.64,1.00)	0.78	(0.62,0.98)
Pregnancy and abortion history								
Nulliparous	Ref.				Ref.			
History of pregnancy, no abortion	0.54	(0.45,0.63)	0.48	(0.40,0.58)	0.59	(0.50,0.70)	0.61	(0.51,0.72)
History of procedural abortion but no medication abortion	1.06	(0.81,1.38)	1.46	(1.13,1.90)	1.16	(0.89,1.52)	1.42	(1.10,1.82)
History of medication abortion	2.22	(1.48,3.34)	2.74	(1.93,3.88)	2.12	(1.38,3.25)	2.66	(1.83,3.86)
Number of barriers trying to access RH services in past 3 years								
None	Ref				Ref.			
One barrier	1.07	(0.88,1.30)	1.42	(1.15,1.77)	1.00	(0.82,1.21)	1.25	(1.03,1.51)
Two barriers	1.16	(0.90,1.49)	1.49	(1.15,1.94)	1.22	(0.95,1.57)	1.58	(1.23,2.03)
3-10 barriers	0.87	(0.71,1.06)	1.50	(1.22,1.85)	0.98	(0.80,1.19)	1.32	(1.09,1.60)
Never tried to access RH services past 3 years	0.45	(0.36,0.57)	0.79	(0.60,1.03)	0.49	(0.39,0.61)	0.62	(0.49,0.79)
Ever experienced medical mistreatment								
None	Ref.				Ref.			
Neglect only (made to feel symptoms ignored/not important)	1.46	(1.22,1.74)	1.28	(1.06,1.56)	1.52	(1.27,1.82)	1.29	(1.07,1.54)
Ridiculed or humiliated, could include neglect	1.56	(1.31,1.86)	1.52	(1.26,1.83)	1.86	(1.57,2.21)	1.70	(1.43,2.01)

aOR=adjusted Odds Ratio; CI=Confidence interval; Ref.=reference group; [a]Total abortion-ban states included AL, AR, LA, IN, KY, MO, MS, ND, OK, SD, TN, and TX; 6 to 18-week ban states included AZ, FL, GA, NC, NE, SC, UT, and WV.
