# Supplementary material for: Changes in Support for Advance Provision and Over-the-Counter Access to Medication Abortion
Source: JAMA Netw Open. 2025 Jan 16;8(1):e2454767. doi: 10.1001/jamanetworkopen.2024.54767 (PMC11739987; doi:10.1001/jamanetworkopen.2024.54767)
Supplement: Supplement 2. — Data Sharing Statement [file jamanetwopen-e2454767-s002.pdf]

Data Sharing Statement

Biggs. Changes in Support for Advance Provision and Over-the-Counter Access to Medication Abortion. *JAMA Netw Open*. Published January 16, 2025.
doi:10.1001/jamanetworkopen.2024.54767

Data
Data available: No

Additional Information
Explanation for why data not available: Data availability statement. Study data are not publicly available due to their sensitive nature and because it could pose a risk to the privacy of participants. The data include information about people's health behaviors, including abortion, which is a sensitive, stigmatized, and sometimes criminalized behavior. Researchers with a legitimate interest in replicating our findings or conducting secondary analyses can contact the corresponding author to discuss the possibility of obtaining anonymized data or aggregate statistics. We will consider all requests on a case-by-case basis and prioritize those that align with the original research objectives and participant consent.
